# Supplementary material for: A Humanized Yeast Phenomic Model of Deoxycytidine Kinase to Predict Genetic Buffering of Nucleoside Analog Cytotoxicity
Source: Genes (Basel). 2019 Sep 30;10(10):770. doi: 10.3390/genes10100770 (PMC6826991; doi:10.3390/genes10100770)
Supplement: Supplementary file 1 [file genes-10-00770-s001.zip › Additional_Files/Additional_File7_GOTermHeatmaps.zip!/Additional_File7_GOTermHeatmaps/multi-eIF_complex.pdf]

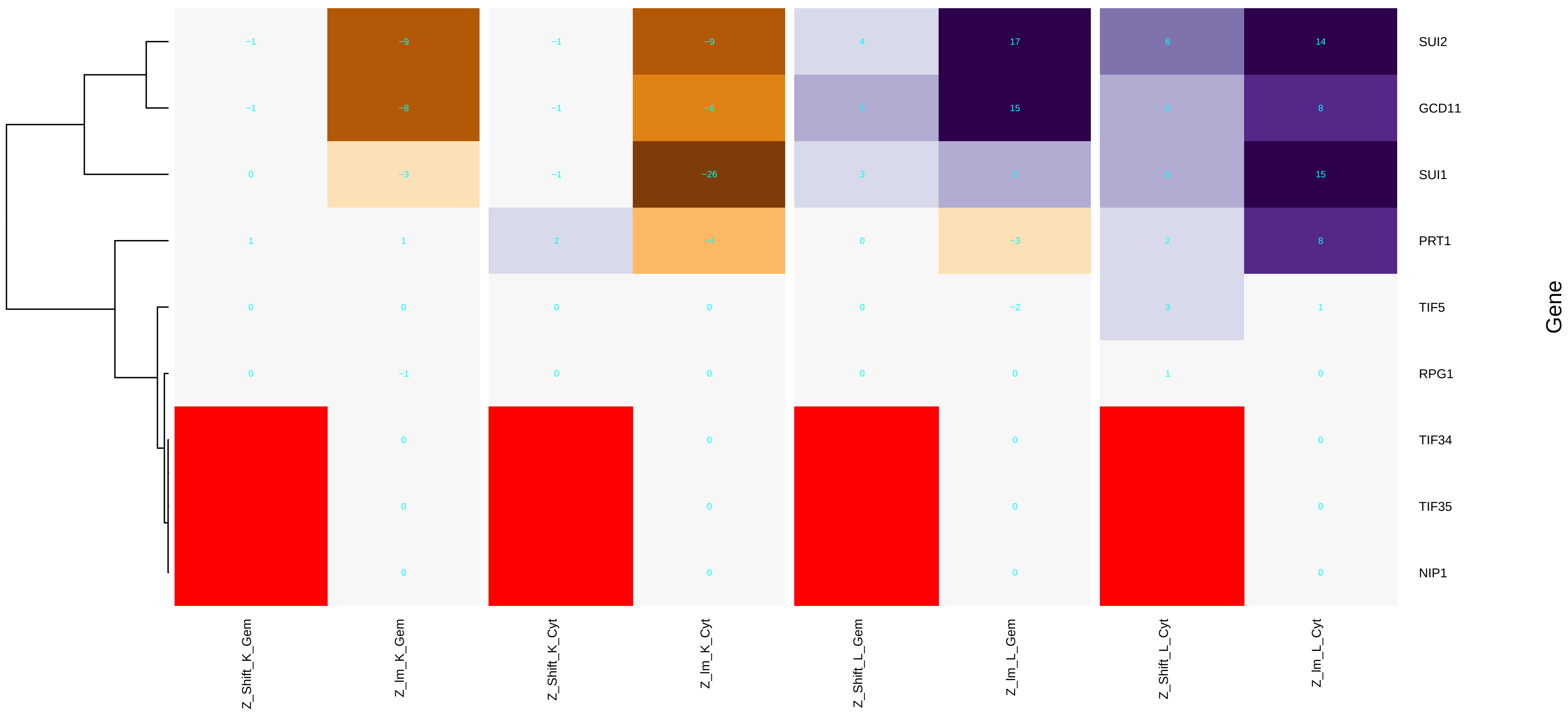

−1
−9
−1
−9
4
17
6
14
−1
−8
−1
−8
5
15
6
8
0
−3
−1
−26
3
5
5
15
1
1
2
−4
0
−3
2
8
0
0
0
0
0
−2
3
1
0
−1
0
0
0
0
1
0
0
0
0
0
0
0
0
0
0
0
SUI2
GCD11
SUI1
PRT1
TIF5
RPG1
TIF34
TIF35
NIP1
Gene
Z_Shift_K_Gem
Z_Im_K_Gem
Z_Shift_K_Cyt
Z_Im_K_Cyt
Z_Shift_L_Gem
Z_Im_L_Gem
Z_Shift_L_Cyt
Z_Im_L_Cyt
